# Nivolumab and Ipilimumab in the Treatment of Metastatic Uveal Melanoma: A Single-Center Experience

**DOI:** 10.1155/2019/3560640

**Published:** 2019-04-17

**Authors:** Vidhya Karivedu, Ihab Eldessouki, Ahmad Taftaf, Zheng Zhu, Abouelmagd Makramalla, Nagla Abdel Karim

**Affiliations:** ^1^Department of Internal Medicine, Division of Hematology/Oncology, University of Cincinnati College of Medicine, Cincinnati, OH, USA; ^2^Department of Biostatistics and Bioinformatics, Department of Environmental Health, College of Medicine, University of Cincinnati, Cincinnati, OH, USA; ^3^Division of Vascular and Interventional Radiology, University of Cincinnati College of Medicine, Cincinnati, OH, USA; ^4^Department of Internal Medicine, Division of Hematology/Oncology, Medical College of Georgia-Augusta University, Augusta, GA, USA

## Abstract

**Background:**

Metastatic uveal melanoma (MUM) is associated with a poor prognosis, with a median overall survival (OS) of 4–15 months. Despite new insights into the genetic and molecular background of MUM, satisfactory systemic treatment approaches are currently lacking. The study results of innovative treatment strategies are urgently needed.

**Patients and Methods:**

This was a retrospective case series of 8 patients with MUM managed at the University of Cincinnati between January 2015 and January 2018. The immune-related Response Evaluation Criteria in Solid Tumors (irRECIST) 1.1 criteria were used for patient evaluation, and magnetic resonance imaging was used for evaluation at treatment checkpoints.

**Objective:**

To assess the clinical outcome of patients with MUM treated with a combination of checkpoint inhibitors.

**Results:**

The series included eight patients, six men and two women, with MUM. Their median age at MUM diagnosis was 69 (range, 55–77) years. All patients were treated with ipilimumab and nivolumab combination along with transarterial chemoembolization (TACE), followed by nivolumab maintenance and monthly TACE procedures. The majority of patients had a partial response or stable disease. Two of the patients had partial response, while four others had stable disease. Two other patients experienced disease progression.

**Conclusion:**

We report the outcomes of eight patients with MUM treated with the combination of ipilimumab and nivolumab. We report the clinical outcome and toxicity associated with this treatment approach. Further studies are warranted to explore immunotherapy in MUM. These findings support the consideration of immunotherapy in MUM.

## 1. Introduction

Uveal melanoma (UM) is the most common primary intraocular malignancy in adults. It accounts for <5% of all melanoma cases in the United States [1, 2]. UM can arise from melanocytes located along the uveal tract, which is the pigmented layer composed of the iris and ciliary body anteriorly and choroid posteriorly. UM is a rare form of melanoma, with an approximate incidence rate of 1,500 new cases diagnosed each year in the US. There is a higher prevalence among Caucasians compared to that in other ethnic groups [3, 4]. Despite effective local therapies, there is a high potential for metastases even after a prolonged period of remission [4, 5]. While the cumulative five- and ten-year metastatic rates reported by the Collaborative Ocular Melanoma Study (COMS) Group were 25% and 34%, respectively, up to 50% of patients develop metastatic disease [6, 7]. The predominant target organ for metastases is the liver (89%). Metastases to the skin, bone, brain, and lungs have also been reported [8]. According to the TNM staging of metastatic disease in uveal melanoma (MUM), M0 is defined as the absence of distant metastasis, while M1a is a disease with distant metastasis with the largest diameter of 3 cm or less, M1b is metastatic disease with the largest diameter of 3.1 to 8 cm, and M1c is metastatic disease with the largest diameter of 8 cm or more [9, 10]. Multiple therapeutic approaches for MUM have been studied but none has shown any impact on the overall survival (OS) [11]. Recent studies have shown that outcomes of patient with MUM are dismal with median overall survival of 12 months from the time of metastasis diagnosis [12]. There is no established standard of care for the systemic therapy of patients with MUM as they are usually excluded from large randomized trials; thus, the current treatment paradigm is based on the National Comprehensive Cancer Network (NCCN) guidelines [13]. Liver-directed therapies such as liver resection in a small subset of patients may induce remission in the setting of single-site metastases potentially prolonging OS, albeit with a high recurrence rate [7, 14]. UM is clinically and biologically distinct from cutaneous melanoma. However, the systemic management of MUM is adapted from that of cutaneous melanoma. Major improvements have followed the introduction of BRAF/MEK inhibitors and immunotherapy in metastatic cutaneous melanoma. Unlike cutaneous melanoma, several studies report lack of BRAF kinase mutations, suggesting lack of benefit from BRAF inhibitors in patients with advanced uveal melanoma [15–17]. Oncogenic mutations in G-protein subunits a (GNAQ) and 11 (GNA11) have been described in 80% of uveal melanomas [18].

Small retrospective studies have reported low response rates to PD-1 inhibitor monotherapy for MUM. The combination of nivolumab and ipilimumab has shown a survival benefit in patients with cutaneous melanoma at the expense of immune-related toxicities and has been approved for the management of metastatic cutaneous melanoma [19–21]. Patients with MUM were excluded from most of the clinical trials; thus, the safety and efficacy of the currently studied combinations remain unclear, especially the ocular toxicity of the combination in patients with MUM [22–24]. Ongoing trials are evaluating the combination of CTLA-4 and PD1 blockade in MUM [25, 26].

In this study, we report our experience in treating patients with MUM with the combination of ipilimumab and nivolumab.

## 2. Case Series

### 2.1. Case 1

**Figure 1:**
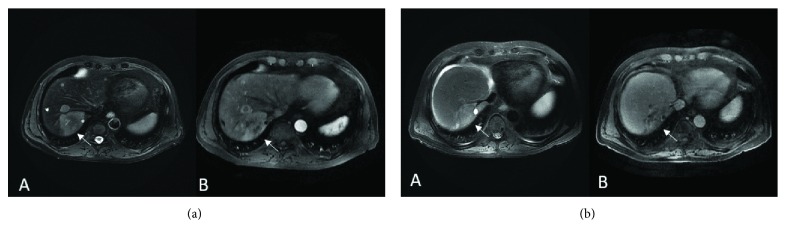
(a) Pretreatment scans (MRI). Axial T2 WI (A) and postcontrast (Eovist) Axial T1 WI (B) showing a 7 cm mass in the right liver lobe (arrows) and multiple smaller lesions in both liver lobes. (b) Posttreatment scans (MRI). Axial T2 WI (A) and postcontrast (Eovist) Axial T1 WI (B) show the decrease in size of the largest mass in the right liver lobe (arrows).

A 68-year-old man was initially diagnosed with right primary choroidal melanoma by histopathology and immunohistochemistry (IHC). He was treated with I-125 plaque brachytherapy in 2013. In April 2016, an abdominal ultrasonography (US) revealed multiple scattered hypodense lesions throughout the liver; the largest lesion was within segment 7 measuring 6.6 × 5.1 cm (M1b). A US-guided liver biopsy confirmed a recurrence, with a lactic acid dehydrogenase (LDH) level of 220 U/L (110-270 U/L) and alkaline phosphatase (ALP) of 22 (7-52 U/L). In April 2016, the patient started a combination of ipilimumab (3 mg/kg) and nivolumab (1 mg/kg) administered every 3 weeks. After three cycles of treatment, imaging revealed the same number of hypodense lesions; the largest lesion measured 5.5 × 3.4 cm (Figure 1). In July 2016, treatment was stopped due to severe autoimmune colitis as a side effect of the immunotherapy. Later that year, in September 2016, the patient continued nivolumab alone (240 mg every 2 weeks), which was also discontinued in February 2017 due to intolerance. Since then, the patient had received transarterial chemoembolization (TACE) for the hepatic lesions. In June 2017, the patient developed progressive disease, with an LDH of 317 U/L and ALP of 426 U/L. The patient was enrolled to hospice care and the patient expired within a month.

### 2.2. Case 2

A 69-year-old man was referred to an ocular oncologist in 2014 due to visual changes in his left eye. He underwent enucleation in 2014 and histopathology showed T3aN0M0 choroidal melanoma. He underwent systemic staging and did not have metastatic disease at the time. Later in April 2016, surveillance imaging showed multiple pulmonary nodules (M1a), which were diagnosed as metastatic disease by right lung lower lobe wedge resection confirmed by IHC (HMB-45 and MART-1), with an LDH of 191 U/L and ALP of 84 U/L. In July 2016, the patient started nivolumab (1 mg/kg) and ipilimumab (3 mg/kg) administered every 3 weeks. Upon completion of four cycles, the treatment was stopped due to autoimmune colitis as a side effect of immunotherapy. Imaging surveillance in September 2016 showed progressive disease, with an LDH of 231 U/L and ALP of 89 U/L, and the patient started treatment with nab-paclitaxel and he continues to have stable disease with no signs of disease progression for 18 months now.

### 2.3. Case 3

A 77-year-old man was referred to an ocular oncologist in 2014 for visual changes in his right eye. He was diagnosed with a choroidal melanoma by histopathology and IHC, treated with I-125 plaque brachytherapy. Surveillance imaging in March 2017 showed liver and pulmonary lesions (M1a), with an LDH of 168 U/L and ALP of 54 U/L. A liver nodule biopsy confirmed the presence of MUM. The patient completed selective internal radiation therapy (SIRT) to the liver metastases in March 2017. In March 2017, the patient also started treatment with nivolumab (1 mg/kg) and ipilimumab (3 mg/kg) every 3 weeks for a total of four cycles, followed by nivolumab maintenance (240 mg). The patient also underwent TACE simultaneously with immunotherapy every 5–6 weeks starting from May 2017. Nivolumab was stopped in March 2018 due to thrombocytopenia, and the patient continued TACE every eight weeks until September 2018 and later discontinued due to no tumor growth. Repeat imaging in February 2019 showed stable disease.

### 2.4. Case 4

A 76-year-old woman was referred to an ocular oncologist in 2014 for visual changes in her left eye and was diagnosed with a ciliochoroidal melanoma by histopathology, treated with I-125 plaque brachytherapy. Surveillance imaging in June 2017 showed multiple liver lesions with the largest measuring 4.5 × 3.5 cm (M1b). A liver biopsy confirmed MUM. The patient started therapy with nivolumab (1 mg/kg) and ipilimumab (3 mg/kg) every 3 weeks for four cycles, followed by maintenance nivolumab (240 mg) every two weeks simultaneously with TACE every 4 weeks. In October 2017, imaging showed stable liver lesions. Imaging surveillance in November 2017 showed the progression of the liver lesions, with an LDH of 466 U/L and ALP of 442 U/L. Nivolumab was discontinued in November 2017, and the patient expired in January 2018.

### 2.5. Case 5

**Figure 2:**
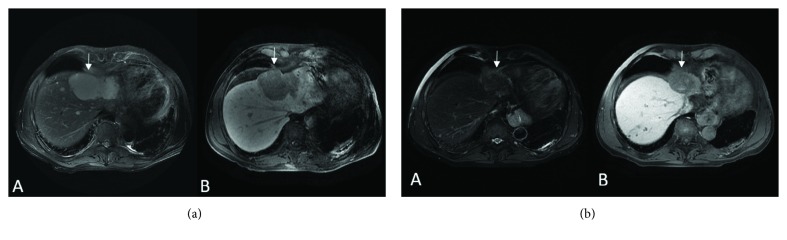
(a) Pretreatment scan (8/22/2016) (MRI). Axial T2 WI (A) and postcontrast (Eovist) Axial T1 WI (B) showing an 11 cm mass in the left liver lobe (arrows) and multiple smaller lesions in both liver lobes. (b) Ongoing treatment (5/2/2018) (MRI). Axial T2 WI (A) and postcontrast (Eovist) Axial T1 WI (B) show the decrease in size of the largest mass in the left liver lobe (arrows) now measuring 4.8 cm with no enhancement. There is a decrease in size and number of the multiple smaller lesions in both liver lobes.

In 2014, a 65-year-old man was referred to an ocular oncologist for a visual change in his left eye and diagnosed with choroidal melanoma by histopathology and IHC, treated with enucleation, T1aN0M0. Surveillance imaging first showed hepatic lesions in January 2016. Active surveillance in August 2016 revealed that his liver lesions had increased in size and number with the largest lesion measuring 7.1 × 5.8 cm (M1b), with an LDH of 641 U/L and ALP of 111 U/L. Liver biopsy confirmed MUM. The patient started therapy with nivolumab (1 mg/kg) and ipilimumab (3 mg/kg) every 2 weeks simultaneously with TACE every 4 weeks in September 2016. A repeated abdominal magnetic resonance imaging (MRI) in November 2016 showed a marked decrease in the size and number of metastatic liver lesions (Figure 2). After four cycles of nivolumab/ipilimumab, he started maintenance nivolumab (240 mg every 3 weeks) in January 2017. Repeat imaging showed continued response until August 2018. Imaging in September 2018 showed progression of disease; therapy switched to nab-paclitaxel. The patient currently has stable disease on nab-paclitaxel and TACE q8 weeks as of March 2019.

### 2.6. Case 6

**Figure 3:**
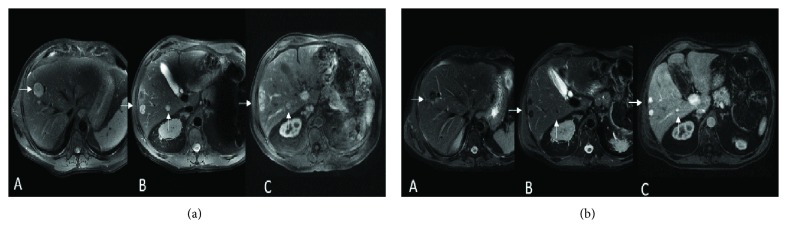
(a) Pretreatment scans (MRI). Axial T2 WI (A, B) and postcontrast (Eovist) Axial T1 WI (C) show multiple metastatic lesions (arrows) in both liver lobes. (b) Posttreatment scans (MRI). Axial T2 WI (A, B) and postcontrast (Eovist) Axial T1 WI (C) show the decrease in size and number of multiple lesions (arrows) in both liver lobes.

A 63-year-old man was initially referred to an ocular oncologist in February 2016 due to a visual change in his left eye. He was diagnosed with ciliochoroidal melanoma by histopathology, T4bN0M0. He was treated with enucleation of his left eye. In February 2017, surveillance imaging showed liver lesions, with the largest measuring 2.2 × 2.1 cm in hepatic segment 7 (M1a) and an LDH of 194 U/L, ALP of 94 U/L; biopsy confirmed metastatic melanoma. The patient started treatment with nivolumab (1 mg/kg) and ipilimumab (3 mg/kg) in May 2017. After two doses of a combination of ipilimumab and nivolumab, he developed colitis, which was treated with prednisone. Repeated imaging in June 2017 showed a decrease in the size of the metastatic hepatic lesion, from 2.2 × 2.1 to 1.7 × 1.5 cm (Figure 3). The patient started nivolumab (240 mg every 2 weeks) in August 2017. In October 2017, imaging showed a mixed response, with stable lesions in segment 7 and new hepatic lesions in segment 8, with an LDH of 242 U/L, ALP of 114 U/L. The patient continued nivolumab until disease progression in April 2018, and the patient expired in June 2018.

### 2.7. Case 7

A 73-year-old woman was referred to an ocular oncologist in June 2015 for visual changes in her right eye, diagnosed with a primary choroidal melanoma by histopathology. She was treated with I-125 plaque brachytherapy in June 2015. Surveillance imaging showed hepatic lesions in September 2015, with LDH of 194 U/L, ALP of 73 U/L. The largest lesion measured 2.2 × 2.2 cm (M1a). A liver biopsy confirmed MUM. She started therapy with nab-paclitaxel and received three cycles simultaneously with TACE for left and right liver lobe metastases. In February 2016, imaging showed disease progression, with LDH of 519 U/L and ALP of 72 U/L. Therefore, the patient started therapy with ipilimumab (3 mg/kg) and nivolumab (1 mg/kg). After one cycle, she developed grade IV myalgia and neuropathy requiring hospitalization and immunotherapy was stopped. In May 2016, the patient was initiated on pembrolizumab simultaneously with monthly TACE procedure for liver metastases. However, she was hospitalized for pulmonary edema and autoimmune hepatitis. Imaging repeated in September 2016 showed the progression of the hepatic lesions. She was later enrolled to hospice care and expired in September 2016.

### 2.8. Case 8

A 55-year-old man was initially diagnosed with primary choroidal melanoma of the left eye in October 2016 by histopathology, treated with I-125 plaque brachytherapy. Surveillance imaging in July 2017 showed numerous liver lesions, the largest measuring up to 1.6 cm (M1a), with an increase in his LDH level to 634 U/L, ALP 65 U/L. A liver biopsy confirmed MUM. He started monthly TACE in August 2017. In September 2017, the patient started therapy with ipilimumab (3 mg/kg) and nivolumab (1 mg/kg) every 3 weeks. He finished his fourth cycle in November 2017. In December 2017, an abdominal MRI showed a mixed response, in which several lesions were stable while others had slightly increased in size, with LDH level of 267 U/L and ALP 256 U/L. The patient later continued maintenance therapy with nivolumab (240 mg) every 2 weeks until January 2018. Repeat imaging in February 2018 showed disease progression and the patient expired in April 2018.

## 3. Discussion

Median OS of MUM patients with M1a disease was 20 months, while M1b disease was 10 months [10]. The current treatment for MUM is based on the recommendations for metastatic cutaneous melanoma. Local interventions such as chemoembolization further guide therapies for MUM. One chemotherapeutic option, dacarbazine, has shown a limited response in MUM [27]. Other chemotherapeutic regimens including temozolomide, cisplatin, treosulfan, fotemustine, and various combinations have been investigated in MUM with similar results [28–30].

Several case series and small prospective studies have evaluated immune checkpoint inhibitors in MUM [31–35]. Ipilimumab, an anti-CTLA4 agent, is an immune checkpoint inhibitor that has shown response rates of 5–10% with ipilimumab in patients with MUM, with a median OS time of 6.0–9.7 months [35–37]. Preliminary data from a phase II trial conducted by the Spanish Melanoma Group (GEM), using front line ipilimumab 10 mg/kg IV every 3 weeks for four doses followed by maintenance doses every 12 weeks until disease progression or acceptable toxicity in treatment-naïve MUM patients, showed promising response rates at a median follow-up time of 5.5 months [38]. However, the Dermatologic Cooperative Oncology Group (DeCOG) conducted an open-label multicenter phase II trial in treatment-naïve or pretreated MUM patients which reported a median progression-free survival (PFS) and OS of only 2.8 and 6.8 months, respectively. Investigators also determined that treatment-naïve patients did not have an improved 1- or 2-year survival compared to previously treated patients [39].

The anti-PD1 agents nivolumab and pembrolizumab have shown greater efficacy in cutaneous melanoma, with an improved side effect profile compared to that of ipilimumab. However, the activity of PD-1 inhibition in UM is not yet well-described. One case series of 10 patients with MUM treated with pembrolizumab reported one complete response (CR), two partial response (PR), and one patient with stable disease (SD) [40]. Another large multicenter case series including 56 patients who received anti-PD1 (nivolumab, pembrolizumab) or anti-PDL1 (atezolizumab) agents showed median OS and PFS of 7.6 months and 2.6 months, respectively [32]. Adverse events should be considered when treating patients with immunotherapy since autoimmune side effects can affect therapy continuation or further management in these patients.

While combination immunotherapy has achieved higher response rates in patients with metastatic cutaneous melanoma compared to those for monotherapy, studies are ongoing to evaluate combination therapy in MUM [25, 26]. Afzal et al. reported a case of MUM treated with the combination of nivolumab and ipilimumab for four cycles, followed by maintenance therapy with nivolumab for two cycles in which the patient achieved a durable response and had continued to do well for 22 months since the start of combination therapy. However, subsequent therapy was stopped due to the development of autoimmune hepatitis [31].

The use of immune checkpoint inhibitors can lead to the development of adverse events and toxicities. The frequencies of immune-related adverse event (iRAE) effects are higher for the combination of PD-1 and CTLA-4 agents compared to the frequencies for any of these therapies alone. Toxicities are common in the GI tract, liver, and skin and the endocrine system. In a phase III trial (CheckMate 067), grade 3 or 4 iRAEs occurred in 55% of the combination group vs. 16% and 27%, respectively, for nivolumab and ipilimumab alone [19].

Diarrhea and colitis are the most frequent iRAEs after ipilimumab either as monotherapy or in combination with PD1 inhibitors (33.1% and 44.1%, respectively). GI symptoms usually appear 6 weeks after treatment initiation. Treatment-related adverse event of any grade leading to their discontinuation happened in 36.4% and 14.8% in the combination arm and ipilimumab group, respectively, with the most common being diarrhea and colitis. In our study, four out of eight patients experienced autoimmune colitis (50%) with the combination. Adverse events were generally manageable with established guidelines, including the use of steroids for grade 3 or 4 adverse events. The safety profile of ipilimumab and nivolumab combination in our study was similar to that observed in cutaneous melanoma receiving combination.

**Figure 4:**
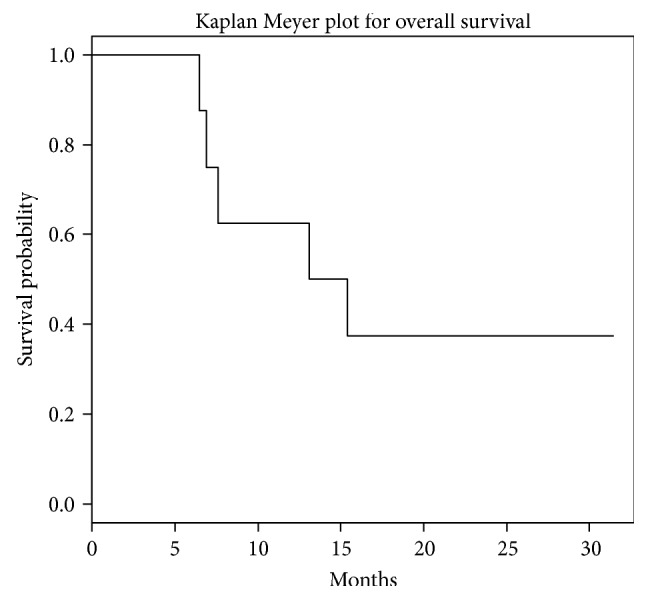
Median overall survival for the entire cohort. Median overall survival for this cohort was 14.2 months.

Our retrospective study assessed patients with MUM treated with immunotherapy combination within a single institute (summarized in Table 1). During the study period, no available open and feasible clinical trials were available to this group of patients. After obtaining written informed consent, immunotherapy was administered on a compassionate use basis since no other appropriate medical therapies were available; no other ethical approvals were needed. In our series, patients were treated with the combination of ipilimumab and nivolumab plus TACE followed by maintenance nivolumab along with monthly TACE procedures. The majority of patients had initial PR or SD. Out of the eight patients, two achieved PR, while four others had SD. Two other patients had progression of disease (POD). Median OS (from the date of immunotherapy initiation to the date of death/date of last follow-up) by Kaplan-Meier methodology for the eight patients was 14 months (Figure 4). The small size of this study limits further analysis, but we were able to see a response, either in the form of SD or PR. Although SD is not considered a response to the drug, it is considered a disease control endpoint as it delays the time to progression. Patients in our study either had M1a or M1b disease. Five of eight patients had M1a disease, OS of these patients from metastatic disease diagnosis to date of death/date of last follow-up ranged 12, 14, 16, 23, and 24 months. The rest of the patients had M1b disease, OS of these patients ranged 7, 15, and 30 months. While previous studies stated that LDH level, CRP level, eosinophil count, and Eastern Cooperative Oncology Group (ECOG) performance status can be used as prognostic factors for UM [41, 42], these data could not be assessed in our study.

TACE depends on the concept of embolization components that can interrupt blood supply to the tumor and thus cause ischemic necrosis and decrease in size. This may lead to controlled growth or even regression of the tumor [43]. TACE was offered to six of the eight patients included in our study and was well-tolerated.

## 4. Conclusion

MUM is associated with a poor prognosis with no current standard of care. There is an urgent need for new strategies for patients with MUM as no therapy has succeeded in improving the OS. Given the development of molecular profiling techniques and the availability of additional immunotherapeutic agents, chemotherapeutic agents, and target therapies, dedicated management strategies and guidelines should be feasible. Immunotherapy in MUM remains an area of active exploration. While checkpoint inhibition with anti-PD-1 and anti-CTLA-4 therapy has drastically changed the treatment approach to cutaneous melanoma, its efficacy in MUM is still being evaluated. Our study reported durable responses in MUM patients treated with anti-PD-1/anti-CTLA-4 therapy; thus, this approach may be a viable option for these patients. Patients who receive combination immunotherapy should also be carefully monitored for iRAEs. The major limitations of this study include its retrospective nature as some of the data could not be retrieved. There is an urgent need for specifically approved systemic and local treatment options for patients with MUM. Given the limited activity of the currently approved agents for advanced melanoma in the treatment of MUM, clinical trials should be performed based on our improved understanding of the biology of this disease. Immunotherapy in UM remains an area of active exploration.

## Figures and Tables

**Table 1:** Summary of cases.

Case #	Age/sex	Primary tumor diagnosis (year), treatment	Diagnosis of metastatic uveal melanoma (MUM), site	Genomic findings	MUM treatment	Side effects	Response to immunotherapy
1	68, M	2013, brachytherapy	April 2016, liver metastases	No genetic alterations	Ipilimumab 3 mg/kg and nivolumab 1 mg/kg for three cycles, followed by nivolumab 240 mg × 2 weeks + monthly TACE until February 2017	Autoimmune colitis	Stable disease
2	69, M	2014, enucleation	June 2016, pulmonary metastases	GNAQ Q209P +ve	Ipilimumab 3 mg/kg plus nivolumab 1 mg/kg every 3 weeks for four cycles	Autoimmune colitis	Progression of disease
3	77, M	2014, enucleation	March 2017, liver and pulmonary metastases	GNA11 Q209L +ve, BAP +ve, MGMT +ve	Ipilimumab 3 mg/kg plus nivolumab 1 mg/kg every 3 weeks for four cycles followed by nivolumab 240 mg every 2 weeks plus monthly TACE	None	Stable disease
4	76, F	2014, brachytherapy	June 2017, liver metastases	GNA11 Q209L +ve	Ipilimumab 3 mg/kg plus nivolumab 1 mg/kg every 3 weeks for four cycles followed by nivolumab 240 mg every two weeks until November 2017	None	Stable August 2017, POD November 2017
5	65, M	2014, enucleation	August 2016, liver metastases	GNAQ Q209P +ve, MYC +ve, BAP +ve, DNMT3A +ve, low mutational burden	Ipilimumab 3 mg/kg plus nivolumab 1 mg/kg every 3 weeks followed by nivolumab 240 mg every 2 weeks plus TACE from January 2017	None	Partial response
6	63, M	2016, enucleation	February 2017, liver metastases	NA	Ipilimumab 3 mg/kg plus nivolumab 1 mg/kg initiated in May 2017 for two cycles, followed by nivolumab 240 mg every 3 weeks plus TACE from August 2017	Autoimmune colitis with combination	Partial response
7	73, F	2015, brachytherapy	September 2015, liver metastases	No genetic alterations, c-KIT +ve	TACE plus Abraxane for three cycles followed by ipilimumab 3 mg/kg plus nivolumab 1 mg/kg for one cycle in February 2017, pembrolizumab 200 mg every 3 weeks from May 2017 to September 2017	Autoimmune colitis with combination	Progression of disease
8	55, M	2016, brachytherapy	June 2017, liver metastases	NA	Monthly TACE from August 2017, ipilimumab 3 mg/kg plus nivolumab 1 mg/kg every 3 weeks for four cycles followed by nivolumab 240 mg every 2 weeks from December 2017	None	Stable disease
