# Corrigendum to “Nivolumab and Ipilimumab in the Treatment of Metastatic Uveal Melanoma: A Single-Center Experience”

**DOI:** 10.1155/2019/3868790

**Published:** 2019-10-28

**Authors:** Vidhya Karivedu, Ihab Eldessouki, Zelia Correa, Ahmad Taftaf, Zheng Zhu, Abouelmagd Makramalla, Nagla Abdel Karim

**Affiliations:** ^1^Department of Internal Medicine, Division of Hematology/Oncology, University of Cincinnati College of Medicine, Cincinnati, OH, USA; ^2^Ocular Oncology Service, Department of Ophthalmology, University of Cincinnati College of Medicine, USA; ^3^Department of Biostatistics and Bioinformatics, Department of Environmental Health, College of Medicine, University of Cincinnati, Cincinnati, OH, USA; ^4^Division of Vascular and Interventional Radiology, University of Cincinnati College of Medicine, Cincinnati, OH, USA; ^5^Department of Internal Medicine, Division of Hematology/Oncology, Medical College of Georgia-Augusta University, Augusta, GA, USA

In the article titled “Nivolumab and Ipilimumab in the Treatment of Metastatic Uveal Melanoma: A Single-Center Experience” [1], Dr. Zelia Correa was missing from the authors' list. Dr. Zelia Correa commented on the article and referred the patient population in the study. The corrected authors' list and affiliations are shown above.
